# Acute Ocular Complications from Self-Administered Topical Kermes

**DOI:** 10.4103/0974-9233.71589

**Published:** 2010

**Authors:** Huda A. Al-Ghadeer

**Affiliations:** Anterior Segment Department, King Khaled Eye Specialist Hospital, Riyadh, Kingdom of Saudi Arabia

**Keywords:** Cicatrization, Conjunctiva, Crimson, Kermes, Ocular Complication, Symblepharon, Visual Impairment

## Abstract

To report severe ocular complications and their management after self-administered topical kermes dye eye drops. A case report of a 55-year-old man who suffered severe ocular surface damage after application of topical kermes eye drops to his left eye. Active compounds of the kermes eye drops were studied for their composition. Patient reported decreased vision in the affected eye and the external eye examination revealed complete corneal abrasion, cicatrization of the conjunctiva, and symblepharon formation. The patient required immediate cleansing of the ocular surface along with irrigation. He was treated with topical corticosteroids and frequent lubrication. Gas chromatography/mass spectrometry analysis of the retrieved topical material revealed the presence of acid. The patient’s visual acuity improved from 20/200 before treatment to 20/25 after treatment. Topically administered kermes eye drops may cause severe ocular injuries. Public education, early recognition of such injuries, and timely intervention may prevent permanent damage to the ocular adnexae.

## INTRODUCTION

Kermes (Arabic: Qirmiz), obtained from the dried bodies of the female of a scale insect (Kermes ilicis) is one of the oldest dyes known to man. In ancient times, Kermes was used either as folk medicine or for cosmetic purposes, such as dyeing hair.^1^ A spectrum of ocular complications arising after the use of topically administered Kermes dye in various forms have been reported in the past.^1^ We report here acute ocular complications after the use of Kermes dye eye drops in an adult patient that has not, to the best of our knowledge, been reported in English peer-reviewed literature.

## CASE REPORT

A 55-year-old man presented to the emergency room with chief complaints of decreased vision, severe pain, tearing, and photophobia of his left eye after administering a drop of Kermes topical eye drops, which he obtained from a homeopathic/folk medicine man. Apart from hypertension, the patient’s medical history was unremarkable.

**Figure 1:**
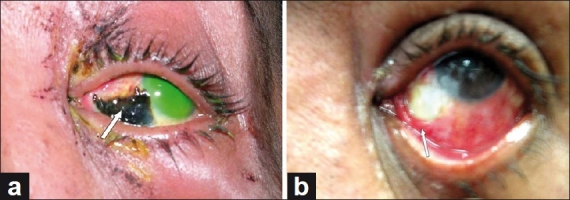
(a) Complete corneal abrasion using topical fluorescein hydrochloride. Note the black elevated lesion (arrow) secondary to the use of kermes (b) Inferior fornix of the left eye shows foreshortening and symblepharon formation (arrow)

Ophthalmic examination revealed best corrected visual acuity of 20/30 in the right eye (OD) and 20/200 in the left eye (OS). Examination of the OD was unremarkable except for mild cataract. Examination of OS revealed swelling and black pigmentation over the upper eyelid. There was conjunctival chemosis along with well-demarcated black elevated areas inferonasally. Evidence of early cicatrization involving the fornix of the conjunctiva and symblepharon formation were present. Some particulate material from the lower fornix was removed and submitted for chemical analysis. Administration of topical fluorescein hydrochloride revealed a large epithelial defect involving the whole cornea [Figure 1a]. There was no evidence of perilimbal ischemia. Intraocular pressure was normal in both eyes.

The left eye was immediately irrigated with normal saline for 30 min. The patient was instructed to frequently lubricate the left eye with preservative-free artificial tears in addition to the administration of topical Ofloxacin 0.3% drops 4 times a day for 1 week and topical prednisolone acetate 1% 4 times a day for the first week then it was tapered. In addition, topical cyclopentolate 1% drops 2 times a day were prescribed for the first 2 weeks. Over the following several days, the patient’s best corrected visual acuity improved to 20/60. Eyelid swelling along with skin discoloration completely resolved. The corneal epithelial defect healed completely without scarring [Figure 1b].

Gas chromatography/mass spectrometry of the submitted sample revealed the main ingredient to be kermesic acid (C_18_H_12_O_9_) consistent with Kermes. In addition to kermesic acid, there were elements of barium, iron, manganese, potassium, and copper. Six months after presentation, symblepharon was still noted, however, the best corrected visual acuity OS was 20/25 without corneal scar.

## DISCUSSION

The prevalence of preventable blindness in the developing countries may be 20 times higher than in the developed countries. This is especially pertinent as the vast majority of blind people reside in the underdeveloped parts of Africa and Asia. There are many causes of visual impairment in countries, such as Saudi Arabia. Many of these causes are preventable through public education and provision of modern health care. Unfortunately, in the rural areas of Saudi Arabia, folk medicine is still actively practiced. The topical use of folk remedies is a common practice in central and southern parts of Saudi Arabia.

Ocular cicatricial conjunctival damage may occur after longterm use of topically administered medications, such as epinephrine, pilocarpine, idoxuridine, timolol, echothiophate iodide (phospholine), and demecarium bromide.^2^–^5^ Our patient demonstrated clinical features similar to those of idiopathic ocular cicatricial conjunctival changes in the form of symblepharon formation in addition to its serious effects on the corneal epithelium with only one application of Kermes eye drops.

Kermes has been used for centuries for different purposes among which include its use as a folk medicine.^1^,^6^,^7^ However, the ocular use of the dye and its associated acute toxicity has been rarely reported. Kermes may injure the conjunctiva through toxicity induced by chemical or thermal injury.^6^ Initial manifestations may be conjunctivitis with irritation, burning, and tearing and may be identical to those of ocular cicatricial pemphigoid. Kermes is an acid and repeated use of Kermes may induce a chronic acid burn of cornea, which may lead to conjunctival cicatrization, symblepharon formation, and keratinization.^6^,^7^ Our patient developed severe acute ocular injuries, including corneal abrasion, and severe conjunctival injury, as well as cicatrization of the conjunctiva with symblepharon formation. Early intervention and treatment resulted in prevention of chronic cicatricial damage and vision loss.

In a previous study, Tabbara^7^ reported an association between cicatricial pseudopemphigoid and the long-term use of topical kermes in 36 patients from Saudi Arabia. The majority of the patients were female between 42 and 76 years of age.^7^ All the patients had used topical kermes prepared at home by dissolving the dye in water and used the solution between 2 and 8 times daily for an average period of 6 years.^7^ Clinically these patients showed evidence of conjunctival cicatrization, obliteration of the fornices, conjunctival epidermization, keratinization of the cornea, dry-eye syndrome, symblepharon formation, and obliteration of the canaliculi. Biopsy specimens obtained during the cataract surgery revealed squamous metaplasia with keratinization and fibrosis.^7^ Additionally, there was a significant loss of conjunctival goblet cells with extensive fibrosis of the substantia propria.^7^ Chemical analysis revealed that the main ingredients of the solution were barium, manganese, potassium, and copper with pH of 4.95 in de-ionized water.^7^ Kermes was found to have antiseptic properties and inhibited the growth of *Escherichia coli and Staphylococcus aureus* at a concentration of 3.2 mg/mL.^7^

In conclusion, immediate and prolonged irrigation of the conjunctiva followed by aggressive early management may be essential to promote ocular surface healing and to provide the best opportunity for visual rehabilitation in patients who present after acute injury from the use of home-made topical folk-medicine remedies, such as Kermes. Ophthalmologists should be aware of the acute side effects of some of these folk remedies, as even a single drop of kermes dye may result in conjunctival scarring similar to that caused by chronic use. Public education programs may raise awareness regarding vision-threatening complications arising from the use of such medications.
